# Genome Sequence of a Marbled Eel Polyoma-Like Virus in Taiwan

**DOI:** 10.1128/genomeA.01607-16

**Published:** 2017-02-09

**Authors:** Chiu-Ming Wen, Ping-Chung Liu, Fan-Hua Nan

**Affiliations:** a Department of Life Sciences, National University of Kaohsiung, Kaohsiung, Taiwan; b Department of Aquaculture, National Taiwan Ocean University, Keelung, Taiwan

## Abstract

We report here the complete genome sequence of a virus isolated from a diseased marbled eel (*Anguilla marmorata*) in Taiwan. The virus has been characterized as being related to Japanese eel endothelial cell-infecting virus (JEECV), with a large T-antigen-like protein. The sequence of the marbled eel virus displays low homology to the JEECV.

## GENOME ANNOUNCEMENT

Marbled eel (*Anguilla marmorata*) is an important emerging species aquaculture in Taiwan (1). Recently, a virus was isolated from the morbid eels which had petechiae throughout the body. The *Anguilla marmorata* virus (AMV) was named marbled eel polyoma-like virus (MEPyV) or *Anguilla marmorata* polyoma-like virus (AMPyV), with a polyomavirus large T-antigen-like (LTL) gene (2). The partial protein sequence has been characterized as being close to the counterpart of Japanese eel endothelial cell-infecting virus (JEECV), which is the pathogen of viral endothelial cell necrosis of eel (2–5). JEECV has a double-stranded circular DNA genome, which suggests that it is a chimeric virus between a polyomavirus and an unknown viral family (6). Additionally, both JEECV and MEPyV have adenovirus-like virions (2, 4). Here, we report the complete genome sequence of MEPyV.

MEPyV (AMV-6) was proliferated in EK-1 cells, and the DNA was isolated and purified by a modification of the Hirt method (7) and delivered to Welgene Biotech (Taipei, Taiwan) for next-generation sequencing. The DNA was further extracted using a WelPrep DNA kit by Welgene and sonicated using a Misonix 3000 sonicator for sizes ranging from 400 to 500 bp, which were performed with a bioanalyzer DNA 1000 chip (Agilent Technologies). One microgram of sonicated DNA was end repaired, A-tailed, and adaptor ligated, according to the Illumina TruSeq DNA preparation protocol, and ConDeTri was implemented for trimming (8). Cleaned and filtered nuclear reads were assembled *de novo* using ABySS (9). All obtained sequences were aligned and manually reassembled.

The circular genomic DNA comprised 16,930 bp and displayed very low similarity to known viruses, including JEECV. A BLASTN search showing the sequence fragment at positions 14627 to 14713 and at 3223 to 3295 was respective homologous to some polyomavirus large T-antigen (LT) genes and certain phycodnaviruses, baculoviruses, and herpesviruses. The genome was suggested to encode 10 proteins, including an LTL, a DNA polymerase, an adenain-like protein, five capsid proteins, and two unknown proteins. Alignment revealed that the LTL amino acid sequence was closer to the LT of giant guitarfish polyomavirus (10) than the LTL of JEECV. Despite the LTL and DNA polymerase, all the MEPyV protein sequences displayed highest homology to JEECV than other known viruses. These data will be helpful to clarify the identity of MEPyV and relationship between MEPyV and JEECV.

### Accession number(s).

The genome sequence of AMV-6 has been deposited in GenBank under the accession no. KX781210.
